# Hemangiopericytoma in the sacrococcygeal space: a case report

**DOI:** 10.1186/1752-1947-4-8

**Published:** 2010-01-14

**Authors:** Yuji Kitahata, Shozo Yokoyama, Katsunari Takifuji, Tsukasa Hotta, Kenji Matsuda, Toshiji Tominaga, Yoshimasa Oku, Takashi Watanabe, Junji Ieda, Hiroki Yamaue

**Affiliations:** 1 Second Department of Surgery, Wakayama Medical University, School of Medicine, Kimiidera, Wakayama, 641-8510, Japan

## Abstract

**Introduction:**

A hemangiopericytoma is a rare, soft-tissue tumor of vascular origin derived from a pericyte of Zimmerman, which is a modified smooth muscle cell that surrounds the small blood vessels. Hemangiopericytomas can occur wherever there are vascular capillaries. However, there are no previous reports of a hemangiopericytoma in the sacrococcygeal space.

**Case presentation:**

We describe the first reported case of a hemangiopericytoma found in the sacrococcygeal space. A 47-year-old Japanese woman presented with a palpable tumor on the left side of her anus. Preoperative imaging indicated that the tumor was in the sacrococcygeal space without invasion of other organs. A complete resection was performed via a parasacral incision. The histological and immunohistochemical staining patterns supported the diagnosis of a hemangiopericytoma.

**Conclusion:**

A complete resection without piecemeal excision is the best way to treat a hemangiopericytoma. Recognizing the presence of a hemangiopericytoma in the sacrococcygeal space requires appropriate surgery.

## Introduction

A hemangiopericytoma is a rare, soft-tissue tumor of vascular origin derived from a pericyte of Zimmerman, which is a modified smooth muscle cell that surrounds the small blood vessels. This type of tumor was first described by Stout and Murray in 1942 [1]. It represents approximately 5% of all sarcomatous tumors, and can occur anywhere, but more usually in the musculature of the extremities, retroperitoneum, pelvis (uterus, ovary, and urinary bladder), head, neck and lungs [2]. There are no reports of a hemangiopericytoma in the sacrococcygeal space. Since the recommended treatment for a hemangiopericytoma is wide excision, due to high local recurrence [3,4], it is important to recognize the presence of this malignant tumor in the area surrounding the anus, where various tumors occur. We describe a rare case of a hemangiopericytoma in the sacrococcygeal space.

## Case presentation

**Figure 1:**
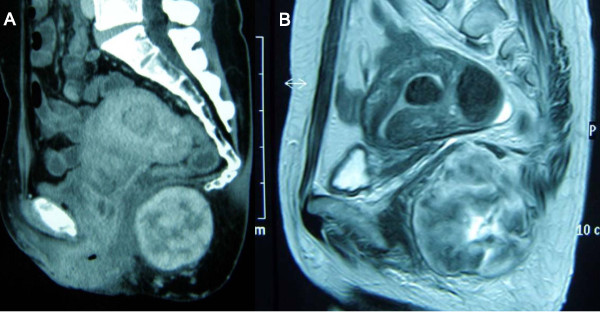
**(A) Computed tomography scan showing a large mass in the sacrococcygeal space**. (B) Magnetic resonance imaging scan showing heterogeneous high intensity in the outer layer and extremely high intensity in the central layer in T2-weighted image.

A 47-year-old Japanese woman presented with a palpable tumor on the left side of her anus. The tumor was elastic and hard and had a smooth surface. She had no pain or tenderness associated with the lesion, and no other clinical symptoms. Computed tomography (CT) scan showed a mass in the sacrococcygeal space with a smooth surface and no invasion to the rectum (Figure 1A). Magnetic resonance imaging (MRI) showed that the outer layer was heterogeneous with high intensity, and that the central layer had an extremely high intensity in T2-weighted images, indicating that the inside of the mass had a rich blood flow (Figure 1B). Rectoscopy revealed that the mucosal surface was intact, and an endoscopic ultrasonography (EUS) demonstrated that the tumor was not derived from the rectum. Fluorodeoxyglucose positron emission tomography (FDG-PET) showed that the maximum standardized uptake value (SUV) at the area was 2.64. The expected SUV is 1.80 to 1.42 in benign soft tissue masses, and 4.20 to 3.16 in malignant soft tissue masses [5].

**Figure 2:**
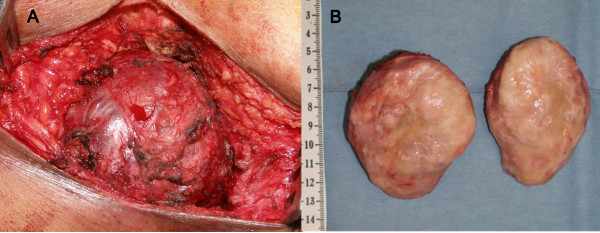
**(A) This is a macroscopic image of the 80 × 75 × 65 mm-sized tumor in the sacrococcygeal space**. (B) This image shows the excised tumor with a capsule, with its cut surface mostly grayish white and partially reddish.

**Figure 3:**
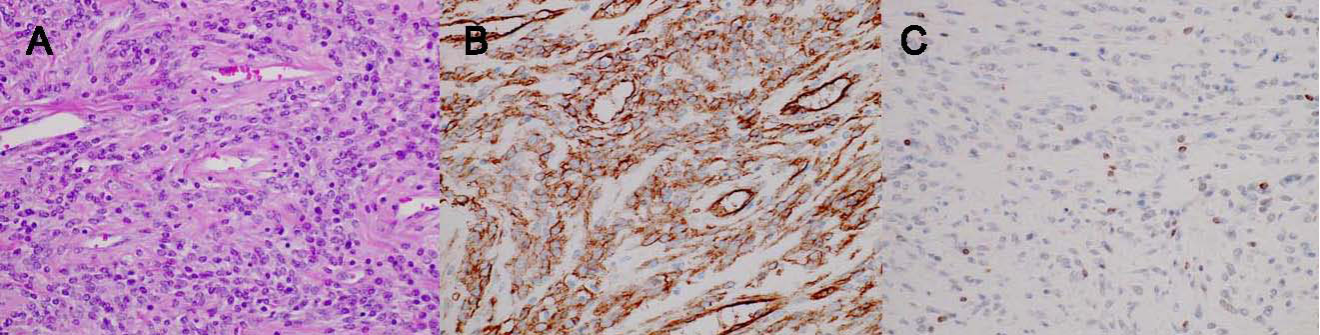
**(A) Hematoxylin and eosin staining revealing spindle-shaped cells surrounding the endothelial-lined vascular spaces**. (B) Immunohistochemistry demonstrating CD34 positive tumor cells. (C) Immunohistochemistry demonstrating Bcl-2 negative tumor cells.

The pre-operative images indicated that the tumor was not derived from the rectum. Because of its vascularity, the pre-operative diagnosis was a soft-tissue tumor such as a solitary fibrous tumor, fibrous histiocytoma, synovial sarcoma, mesenchymal chondrosarcoma, or hemangiosarcoma. A biopsy was avoided due to the risk of needle track seeding. The patient underwent a tumorectomy via parasacral incision, without a rectectomy, because pre-operative examinations including a CT and EUS revealed that the rectum was intact. The tumor was completely removed (Figure 2A). The excised tumor was 80 × 75 × 65 mm in diameter with a capsule. Its cut surface was mostly grayish-white and partially reddish (Figure 2B). Histopathological features of the hematoxylin and eosin staining revealed that the tumor contained spindle-shaped cells surrounding the endothelial-lined vascular spaces, which is consistent with the histology of hemangiopericytoma (Figure 3A). Argyrophil fibers were seen on silver impregnation surrounding the tumor cells.

We performed an immunohistological analysis to obtain a diagnosis of the type of mesenchymal tumor. The mesenchymal tumor cells in our patient stained positive for CD34 (Figure 3B) and vimentin, and negative for Bcl-2 (Figure 3C), CD99, c-kit, factor VIII, desmin, alpha-smooth muscle actin, S-100 protein, epithelial membrane antigen, and keratin. The mitotic rate was 1 per 10 high-power fields. No necrotic lesion was observed in our patient's tumor. The tumor was pathologically diagnosed as a hemangiopericytoma.

## Discussion

Little has been published about hemangiopericytoma, a rare, soft-tissue tumor. It can occur anywhere vascular capillaries are found. The tumors most commonly occurs in the musculature of the extremities, retroperitoneum, pelvis (uterus, ovary, and urinary bladder), head, neck and lungs [3,4].

The pathological diagnosis of a hemangiopericytoma, in comparison to other mesenchymal tumors such as solitary fibrous tumors, can be difficult [6]. Bcl-2 and CD99 immunohistochemistry were used to distinguish a hemangiopericytoma from a solitary fibrous tumor because a solitary fibrous tumor is positive for Bcl-2 [7] and CD99 [8]. We diagnosed a hemangiopericytoma following an examination of the structural features of the mass. Spindle-shaped cells surrounding the endothelial-lined vascular spaces were observed by hematoxylin and eosin staining, and the mass was positive for CD34 and vimentin and negative for Bcl-2, CD99, c-kit, factor VIII, desmin, alpha-smooth muscle actin, S-100 protein, epithelial membrane antigen, and keratin upon immunohistochemical analysis. Making a differential diagnosis between a solitary fibrous tumor and a hemangiopericytoma is particularly difficult and controversial [9], and a novel molecular marker for distinguishing between the two close entities is required.

Radiotherapy and chemotherapy are not generally effective for the treatment of a hemangiopericytoma [10]. Some have advocated the use of adjuvant radiotherapy in response to the locally aggressive nature of hemangiopericytomas but, due to tumor radioresistance, no differences in local disease control were observed between treatment with and without adjuvant radiotherapy [11]. Spitz *et al. *reported that hemangiopericytomas showed a poor response to chemotherapy. They treated six patients with pre-operative chemotherapy, and only one of them responded to anthracycline-based chemotherapy [3]. Therefore, complete surgical resection is the only effective therapy for hemangiopericytoma.

Spitz *et al. *also reported that 5-year and 10-year survival rates of patients with a hemangiopericytoma were 71% and 54%, respectively. In addition, they noted that the survival rate differed between a curative and a non-curative resection. The 5-year survival rate in patients treated with curative resection and non-curative resection was 79% and 50%, respectively [3]. These data indicate that a complete resection is necessary to improve patients' survival rates. Since many benign and malignant diseases occur in the area surrounding the anus, recognizing the presence of this malignant tumor in the sacrococcygeal space is important in order for an anorectal surgeon to avoid inappropriate surgery such as piecemeal excision.

## Conclusion

This report presented a rare case of a hemangiopericytoma in the sacrococcygeal space. Many diseases are associated with anal lesions, therefore a thorough differential diagnosis and complete resection without piecemeal excision must always be performed in the management of this type of malignant tumor.

## Abbreviations

CT: computed tomography; FDG-PET: fluorodeoxyglucose positron emission tomography; MRI: magnetic resonance imaging; SUV: standardized uptake value.

## Consent

Written informed consent was obtained from the patient for publication of this case report and any accompanying images. A copy of the written consent is available for review by the Editor-in-Chief of this journal.

## Competing interests

The authors declare that they have no competing interests.

## Authors' contributions

YK initiated the concept of the case report, performed the literature search, and wrote the manuscript. SY performed the pathological investigations, helped in the literature search, and approved the final write up. KT performed the surgery. TH and KM helped revise the manuscript. TT assisted in the surgery and also helped draft the manuscript. YO and TW helped revise the manuscript. JI helped revise the article. HY contributed to the clinical management of the patient and helped revise the manuscript. All authors read and approved the final manuscript.
